# Hemolytic Activity of Vaginal *Candida albicans* Isolates and Antifungal Effects of Quinalizarin with Hemolysis Modulation

**DOI:** 10.3390/pathogens15040401

**Published:** 2026-04-08

**Authors:** Monika Janeczko, Elżbieta Kochanowicz

**Affiliations:** Department of Molecular Biology, Faculty of Medicine, The John Paul II Catholic University of Lublin, 20-708 Lublin, Poland; elzbieta.kochanowicz@kul.pl

**Keywords:** *Candida albicans*, candidalysin, *ECE1*, hemolysis, quinalizarin, vulvovaginal candidiasis

## Abstract

This study evaluated the hemolytic activity of *Candida albicans* isolates from the female reproductive tract and investigated the in vitro effects of quinalizarin on fungal growth, hemolysis, and *ECE1* expression. Ninety-four clinical *C. albicans* isolates and three ATCC reference strains were analyzed. Hemolytic activity was quantified in culture supernatants and normalized per 10^7^ cells. Antifungal susceptibility and the effect of quinalizarin on hemolysis were assessed using broth microdilution and hemolysis assays. Expression of the *ECE1* gene was evaluated by quantitative real-time PCR in three selected hemolytic strains. Drug interactions between quinalizarin and fluconazole were determined using the fractional inhibitory concentration index (FICI). Among the 97 tested strains, 78 exhibited hemolytic activity with variable intensity. Quinalizarin demonstrated antifungal activity, with MIC values ranging from 2 µg/mL to 256 µg/mL, and showed synergistic effects with fluconazole in selected strains. Exposure to quinalizarin at subinhibitory concentrations reduced *ECE1* transcript levels to 22.8–73.6% of controls (*p* < 0.05) in the analyzed strains. However, the phenotypic effect on hemolysis was limited, with residual activity remaining high: 82% (*p* < 0.05), 93.7% (*p* < 0.05), and 83% (*p* < 0.05) relative to untreated controls in *C. albicans* ATCC 10231, ATCC 90028, and a clinical isolate, respectively. FICI analysis confirmed synergistic interactions between quinalizarin and fluconazole. This preliminary in vitro study highlights the need for further investigation into the relationship between *ECE1* expression, candidalysin-mediated damage, and the antifungal potential of quinalizarin.

## 1. Introduction

According to the World Health Organization (WHO), fungal infections represent a significant global health burden, affecting over one billion individuals and contributing to an estimated 3.8 million deaths annually, a toll that exceeds that of tuberculosis and malaria combined [1]. Among the most common fungal pathogens responsible for both mucosal and systemic infections is *Candida albicans*, a polymorphic opportunistic yeast that normally colonizes the human skin, oral cavity, gastrointestinal tract, and genitourinary tract without causing disease in immunocompetent hosts. However, disruptions in epithelial barriers, alterations in the microbiota, or host immune suppression can promote fungal overgrowth and transition to pathogenicity [2,3].

Mucosal candidiasis, including vulvovaginal candidiasis (VVC) and oral candidiasis, represents the most frequent form of *Candida*-associated disease. Vulvovaginal candidiasis, primarily caused by *C. albicans* (over 90% of cases), affects approximately 75% of women at least once in their lifetime, with 40–50% experiencing recurrent episodes [4]. Recurrent VVC (RVVC), defined as three or more episodes per year, affects approximately 5–8% of women globally [5]. Unlike systemic candidiasis, which typically occurs in immunocompromised individuals, VVC commonly develops in immunocompetent women, with risk factors including pregnancy, antibiotic or corticosteroid use, hormonal contraceptives, and diabetes mellitus [6].

The pathogenicity of *C. albicans* in VVC is mediated by a broad spectrum of virulence factors, including morphological plasticity (yeast-to-hyphae transition), adhesion to epithelial surfaces, biofilm formation, metabolic adaptability, and secretion of exoenzymes, such as phospholipases, esterases, proteases, and hemolysins [3]. Hemolytic activity enables *C. albicans* to lyse erythrocytes and access iron, a critical nutrient for fungal proliferation and dissemination. In humans, the majority of iron is sequestered intracellularly, either bound to ferritin or incorporated into heme-containing compounds. Consequently, pathogens have evolved hemolysin-mediated mechanisms to lyse host cells and release heme-associated iron. This strategy becomes particularly relevant during menstruation, when increased hemolytic activity may facilitate iron acquisition. Therefore, hemolysin represents a key virulence factor essential for the survival and pathogenicity of *C. albicans* in vaginal candidiasis [7,8,9].

Recent studies have demonstrated that hemolytic activity in *C. albicans* is mediated by candidalysin, the first characterized cytolytic peptide toxin in any human pathogenic fungus [10]. Candidalysin is a 31-amino acid amphipathic peptide derived from the *ECE1* gene product through proteolytic cleavage by Kex1/Kex2 proteases. This toxin, secreted during hyphal growth, fulfills multifaceted roles in host–pathogen interactions. Candidalysin directly damages epithelial membranes via pore formation and simultaneously activates host immune responses, including the induction of pro-inflammatory cytokines (e.g., IL-1β, IL-6, IL-8), alarmins, and inflammasome signaling [11,12]. Moreover, candidalysin seems to play a more critical role than fungal load and hyphal formation in the rapid activation of mitochondrial reactive oxygen species (mtROS) in epithelial cells, contributing to cell damage. mtROS have also been identified as central mediators of the vaginal epithelial response to *C. albicans* infection [10,12,13,14].

VVC can be prevented or treated using probiotics and/or azoles. Nevertheless, a complete cure is not always achieved, and treatment can be complicated by antifungal resistance. Women frequently experience recurrent infections even after antifungal therapy, and management of RVVC often requires long-term suppressive azole regimens. Furthermore, while current antifungal agents effectively control acute infections, they provide limited preventive efficacy, particularly in RVVC, underscoring the need for alternative therapeutic strategies [15,16,17]. Given the importance of hemolysis in *C. albicans* pathogenicity, neutralization of candidalysin or modulation of its downstream inflammatory responses has been proposed as a potential therapeutic approach to mitigate immunopathology and symptom development during VVC [7].

In this study, the hemolytic activity of ninety-four clinical isolates obtained from the female reproductive tract and three reference *C. albicans* strains was investigated. The potential therapeutic value of quinalizarin (1,2,5,8-tetrahydroxyanthraquinone) was further assessed by evaluating its effects on fungal growth, *ECE1* gene expression, and the reduction in hemolysis in selected strains. In addition, the interaction between quinalizarin and fluconazole was examined to determine the feasibility of combination therapy using these two antifungal agents.

Quinalizarin, an anthraquinone derivative with well-established anticancer activity, has recently emerged as a promising antifungal agent [18]. Previous studies have demonstrated that quinalizarin exhibits broad-spectrum antifungal activity against opportunistic pathogens, including *Candida* spp. and *Geotrichum capitatum*. Notably, it has been shown to inhibit key virulence traits of *C. albicans*, such as hyphal formation and biofilm development, and to disrupt established biofilms. Its antifungal effects have also been linked to the induction of apoptosis and necrosis, increased intracellular reactive oxygen species levels, and disruption of mitochondrial membrane potential in fungal cells. Importantly, prior research indicated that quinalizarin can suppress the expression of virulence-associated genes, including *ECE1*, which encodes candidalysin, a pivotal factor in host cell damage and hemolysis. These findings provided a compelling rationale to investigate an additional virulence mechanism, specifically hemolysis, in the context of *ECE1* downregulation. Accordingly, the present study aims to advance our understanding of hemolysis-associated virulence in *C. albicans* and to evaluate whether quinalizarin-mediated suppression of *ECE1* expression translates into reduced hemolytic activity. By integrating previous evidence on gene expression modulation with functional virulence outcomes, this study offers novel insights for the development of targeted antifungal strategies, with a particular emphasis on *C. albicans*-associated hemolysis in the context of VVC.

## 2. Materials and Methods

### 2.1. Chemicals and Microbiological Media

Quinalizarin and fluconazole (dissolved in DMSO at a concentration not exceeding 1% in all assays), glucose, fetal bovine serum (FBS), phosphate-buffered saline (PBS), and other reagents were purchased from Sigma-Aldrich (St. Louis, MO, USA). Spider medium (1% nutrient broth, 1% mannitol, 0.2% K_2_PO_4_, 10% fetal bovine serum, pH 7.2), Sabouraud Dextrose medium (SD, Biocorp, Warsaw, Poland), Sabouraud Dextrose Agar medium (SDA, Biocorp, Warsaw, Poland), and RPMI-1640 medium (with L-glutamine, without sodium bicarbonate) (Sigma-Aldrich, St. Louis, MO, USA) were used as well. RPMI-1640 medium was buffered with 0.165 M MOPS (3-(N-morpholino)propanesulfonic acid) to pH 7.0. Human blood for hemolytic tests was purchased from Biomaxima (Lublin, Poland).

### 2.2. Candida albicans Strains

Three strains of *Candida* were obtained from the American Type Culture Collection (ATCC): *C. albicans* ATCC 10231, *C. albicans* ATCC 90028, and *C. albicans* ATCC 90029. Ninety-four clinical strains of *C. albicans* were obtained as stock cultures from the Jan Boży Independent Public Provincial Hospital in Lublin, Poland. The isolates were originally obtained from patients with symptomatic vulvovaginal candidiasis during routine diagnostic procedures. The authors were not involved in the collection of clinical material and therefore had no access to detailed patient metadata (e.g., age, clinical classification, or sampling timeframe). All isolates were anonymized prior to their use in this study. The strains were identified using VITEK 2 YST IC CARDS (bioMérieux, Warsaw, Poland). Stock cultures were stored at −70 °C, sub-cultured on SDA medium, and stored at 4 °C. The fungal strain cultures were routinely maintained in SD medium at 30 °C.

### 2.3. Production of Hemolytic Factor

*C. albicans* isolates were precultured in Sabouraud Dextrose liquid medium at 37 °C for 18 h. Then, the cultures were harvested by centrifugation (1000× *g* for 15 min) and washed with sterile phosphate-buffered saline (PBS). The yeast suspension (10^4^ cells/mL) was inoculated in liquid RPMI-1640 medium supplemented with 3% glucose and incubated at 37 °C for 48 h at 180 rpm. Following growth, cells were harvested by centrifugation at 1000× *g* for 15 min. The supernatants obtained were concentrated tenfold using a centrifugal filter (VWR International, Radnor, PA, USA). The culture supernatant and human erythrocytes suspended in RPMI medium (1 × 10^8^ cells/mL) were mixed at a 1:1 (*v*/*v*) ratio and incubated at 37 °C for 18 h. The positive control consisted of erythrocytes lysed with 1% Triton-X (100% hemolysis), and the negative control comprised a suspension of human erythrocytes in RPMI-1640 medium with PBS buffer at a 1:1 (*v*/*v*) ratio. After incubation, the samples were centrifuged at 1000× *g* for 2 min. Absorbance of the supernatant was determined at 405 nm. Hemolysis was calculated according to the equation Hemolysis (%) = 100 − [(*P* − *S*)⁄(*P* − *N*) × 100)], where *P* is the absorbance of the positive control, *S* is the absorbance of the test sample, and *N* is the absorbance of the negative control. The assay was conducted in duplicate on two separate occasions for each isolate tested. The efficiency of hemolytic factor production was calculated according to the equation *E* = *H*⁄yeast number × 10^7^, where *E* is the efficiency of hemolytic factor production, and *H* is the percentage of hemolysis [19]. Using the same procedure, the effect of quinalizarin on the hemolytic activity of two *Candida* strains, reference *C. albicans* ATCC 90028 and a clinical strain of *C. albicans* 50, was examined. Quinalizarin was used at 4 µg/mL.

### 2.4. Antifungal Susceptibility Assay

Antifungal susceptibility testing was performed according to the Clinical and Laboratory Standards Institute (CLSI) guidelines for broth microdilution CLSI-M27-A3 [20] and as described by Khabnadideh et al. [21]. Quinalizarin was used in a concentration range from 1 µg/mL to 1024 µg/mL. Serial twofold dilutions were prepared exactly as described in the CLSI document. The spectrophotometric method of inoculum preparation, an inoculum concentration of 1.5 × 10^3^ cells/mL, and RPMI-1640 were used. One hundred microliters of yeast inoculum was added to each well of the microdilution plates. Drug-free and yeast-free controls were included. The plates were incubated at 37 °C for 48 h. The MIC was defined as the lowest concentration of the tested compound that inhibited visible microbial growth.

### 2.5. Relative Quantification by Real-Time Reverse Transcriptase qRT-PCR

The expression of the *ECE1* gene was assessed using the real-time PCR technique following quinalizarin treatment. The *C. albicans* cells were treated with 4 µg/mL of quinalizarin or 1% DMSO (control) during propagation in liquid culture in Spider medium supplemented with 10% fetal bovine serum (FBS). After 4 h incubation at 37 °C, the cells were collected, and total RNA was isolated using the YeaStar RNA kit (Zymo Research, Irvine, CA, USA) in accordance with the manufacturer’s instructions. Next, cDNA was synthesized with the use of a Smart First Strand cDNA Synthesis Kit (EurX, Gdańsk, Poland) as specified in the manufacturer’s instructions. TaqMan gene expression assays (Lot: 170255, designed by the manufacturer, ThermoFisher Scientific, Altrincham, UK) and the Fast Probe qPCR Master Mix (EurX, Poland) were used for the PCR detection of transcripts. The cDNA samples were first pre-treated with uracil-N-glycosylase at 37 °C for 2 min to degrade any dUMP-containing PCR products. Next, they were subjected to the initial denaturation process at 95 °C for 3 min, 40 amplification cycles with denaturation at 95 °C for 10 s, and annealing/extension at 60 °C for 30 s with the use of QuantStudio3 (Applied Biosystems, Waltham, MA, USA). The 2^−∆∆Ct^ method was used for the calculation of the relative level of expression of the analyzed genes using *ACT1* as a reference gene [22].

### 2.6. Determination of Fractional Inhibitory Concentration Index (FICI)

The interactions between quinalizarin and fluconazole against the *C. albicans* species were determined using the chequerboard microdilution method and included the determination of the MICs of each drug alone. Two-fold serial dilutions of the drugs were used for MIC determinations. The first antifungal of the combination was serially diluted along the ordinate, while the second drug was diluted along the abscissa. The resulting chequerboard comprised each combination of the two antifungals, with the tubes containing the highest concentration of each antifungal at opposite corners. The plates were incubated at 37 °C, and the MIC end points were read after 48 h. The MIC value was determined as the lowest concentration of the drugs (alone or in combination) that inhibited growth by 100%, compared with that of the drug-free wells. The interaction was classified on the basis of the fractional inhibitory concentration index (FICI). The FICI was calculated using the formula FICI = FIC_A_ + FIC_B_ = (MIC_AB_/MIC_A_) + (MIC_BA_/MIC_B_), where MIC_AB_ is the minimum inhibitory concentration (MIC) of drug A (fluconazole) tested in combination; MIC_A_ is the MIC of drug A tested alone; MIC_BA_ is the MIC of drug B (quinalizarin) tested in combination; and MIC_B_ is the MIC of drug B tested alone. Synergy was defined as a FICI ≤ 0.5, indifference as a FICI between >0.5 and ≤4, and antagonism as a FICI >4. All experiments were performed in triplicate [23].

### 2.7. Statistical Analysis

All data are expressed as a mean ± SD (standard deviation) of three independent experiments. Statistical significance between the treated and control groups was analyzed by Student’s *t*-test using GraphPad Software version 9.1.1 (San Diego, CA, USA). Differences between samples treated with quinalizarin at various concentrations and untreated controls were considered significant when *p* < 0.05.

## 3. Results

This study focused on a recently identified property of candidalysin, namely hemolytic activity, and its potential role in the pathogenesis of *C. albicans* infections of the female reproductive tract. The research was conducted in two stages. In the first stage, the hemolytic properties of *C. albicans* vaginal isolates obtained from gynecological patients were evaluated. In the second stage, the effects of quinalizarin on fungal growth, hemolytic activity, and *ECE1* gene expression were investigated. Additionally, interactions between quinalizarin and fluconazole, one of the most commonly used drugs in VVC, were assessed using the FICI to determine their potential for combined antifungal therapy.

**Figure 1:**
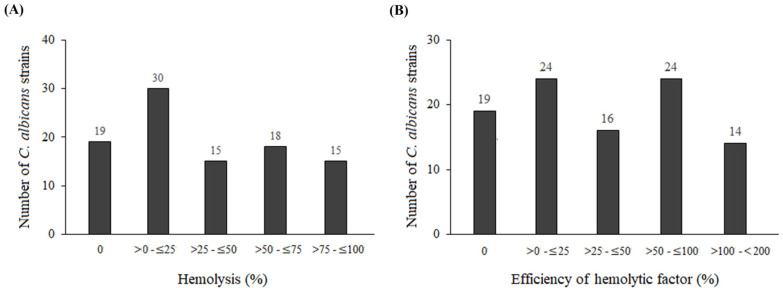
Differentiation of *Candida* strains according to (**A**) the level of hemolysis; (**B**) the efficiency of the hemolytic factor.

As shown by the analysis of post-culture supernatants, 78 *C. albicans* strains, including clinical isolates and reference strains *C. albicans* ATCC 10231, *C. albicans* ATCC 90028, and *C. albicans* ATCC 90029, exhibited hemolytic activity. Among the hemolytic active strains, 30 showed hemolysis levels of 0–25%, 15 of 25–50%, 18 of 50–75%, and 15 isolates exhibited the highest activity, reaching 75–100% (Table 1 and Figure 1A). Hemolytic efficiency normalized to 10^7^ cells also varied between isolates, with 14 strains demonstrating efficiency exceeding 100% (Figure 1B).

**Figure 2:**
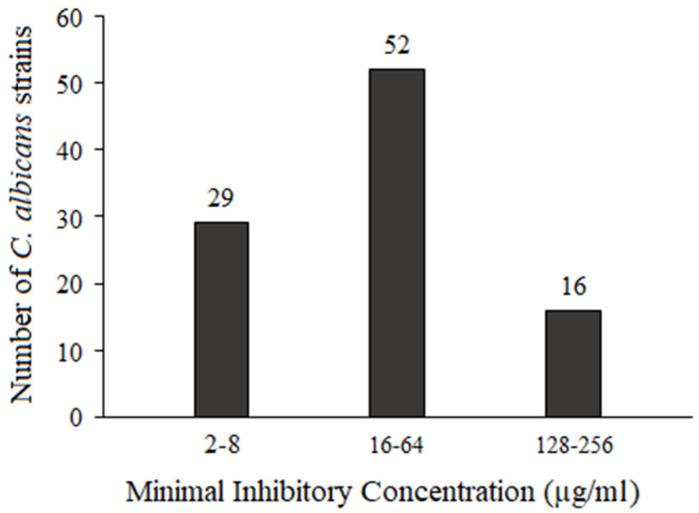
Distribution of MIC values against all tested *C. albicans* strains.

The susceptibility of all *C. albicans* strains to quinalizarin was evaluated. As shown in Table 2 and Figure 2, the minimum inhibitory concentrations (MICs) ranged from 2 to 256 µg/mL. Twenty-nine isolates exhibited high susceptibility (MIC 2–4 µg/mL), 52 displayed intermediate susceptibility (MIC 16–64 µg/mL), and 16 were the least susceptible (MIC 128–256 µg/mL).

**Figure 3:**
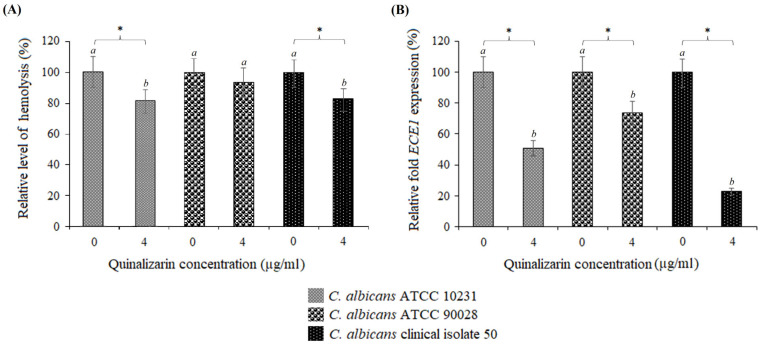
Effect of quinalizarin on hemolytic activity in *Candida* spp.: (**A**) Hemolysin secretion in *C. albicans* ATCC 10231, *C. albicans* ATCC 90028, and clinical isolate 50 after treatment with quinalizarin. (**B**) Relative *ECE1* expression measured by qRT-PCR in the same strains following treatment with quinalizarin. Expression levels are shown as percentages relative to untreated controls. Data represent the mean ± SD from three independent experiments. * *p* < 0.05 was considered statistically significant. Different letters (*a* and *b*) above the error bars indicate significant differences between groups (*p* < 0.05). Groups sharing the same letter are not significantly different.

To investigate the effect of quinalizarin on hemolytic activity and *ECE1* expression, three *C. albicans* strains were selected: *C. albicans* ATCC 10231, *C. albicans* ATCC 90028, and a clinical vaginal isolate (strain 50). Quinalizarin at 4 µg/mL exhibited only a weak inhibitory effect on hemolysis in all tested strains. In *C. albicans* ATCC 10231, hemolytic activity decreased to 82% of the control level. In *C. albicans* ATCC 90028, hemolysis remained at 93.7% of control levels, while strain 50 showed reductions to 83% at the same concentration (Figure 3A). In turn, the qRT-PCR analysis revealed that quinalizarin significantly downregulated *ECE1* expression, reducing transcript levels to 50–73.5% in the reference strains and to as low as 23% in clinical isolate 50 (Figure 3B).

Finally, to explore the therapeutic potential of quinalizarin in combination therapy with fluconazole for VVC, the FICI values demonstrated synergistic interactions between these drugs in all three tested *C. albicans* strains (Table 3).

## 4. Discussion

Vulvovaginal candidiasis (VVC) is one of the most common fungal infections of the mucosa. Despite the availability of azole-based antifungal agents, recurrent infections (RVVC) remain a significant clinical and socioeconomic burden, primarily due to fungal persistence in the female genital tract, complex host–pathogen interactions, and the emergence of antifungal resistance [24]. Accumulating evidence indicates that the immunopathogenesis of VVC is not solely related to fungal proliferation but is largely driven by an exaggerated host inflammatory response induced by hyphal virulence factors. Among these, candidalysin has emerged as a central mediator of mucosal damage and inflammation. Experimental infections with *C. albicans* mutants lacking the *ECE1* gene or expressing truncated forms of candidalysin lead to a marked reduction in tissue damage, neutrophil recruitment, and cytokine production, despite normal hyphal formation [11,25,26]. In addition to its cytolytic and proinflammatory properties, candidalysin also exhibits hemolytic activity, contributing to nutrient acquisition, including iron, from host erythrocytes [12,27].

This study analyzed the hemolytic activity of *C. albicans* isolates from the female reproductive tract and examined the inhibitory potential of quinalizarin on this virulence trait and *ECE1* gene expression. Previous work demonstrated the antifungal activity of quinalizarin, which was associated with reduced hyphal growth, inhibition of biofilm formation, and disruption of mature *C. albicans* biofilms [18]. In the present study, the understanding of its therapeutic potential was further expanded. Furthermore, the interaction between quinalizarin and fluconazole was evaluated to assess their potential in combined antifungal therapy in VVC.

The vast majority of clinical *C. albicans* isolates (approximately 80%) exhibited hemolytic activity, indicating that this virulence factor is common among vaginal strains. These results are consistent with Nouraei et al. (2023), who reported that 90 of 119 isolates (75.6%) obtained from cases of vaginal candidiasis exhibited hemolytic activity [28]. The study also revealed substantial variation in hemolytic levels among the isolates. The largest group (30 strains) showed hemolysis levels not exceeding 25%, whereas 15 strains exhibited activity ranging from 75% to 100%. Such wide variability suggests that hemolytic mechanisms are likely regulated in a strain-dependent manner, possibly due to differences in *ECE1* expression, candidalysin secretion, or hyphal formation efficiency. Interestingly, some isolates displayed hemolytic activity exceeding 100% after normalization to cell count, indicating that erythrocyte lysis may actively contribute to nutrient acquisition during vaginal mucosal infection. It should be noted that, while candidalysin has been identified as a major hemolytic and cytolytic toxin in *C. albicans*, we did not directly measure peptide levels in the isolates tested in this study. Therefore, attributing hemolytic activity solely to candidalysin in these clinical strains remains a hypothesis rather than a confirmed mechanism. Similarly, the suggestion that strains with higher hemolytic activity may be more aggressive, whereas strains with lower hemolytic activity may favor persistent colonization, is plausible based on the literature [10,29,30,31,32] but remains speculative in the absence of clinical severity, recurrence, host factors, or in vivo data. These interpretations are presented here as hypotheses for future investigation rather than definitive conclusions. Given that candidalysin has been identified as a principal toxin responsible for both cytolytic and hemolytic activity, our findings are consistent with the hypothesis that variable candidalysin activity, and consequently differential hemolysis, may underlie the distinct clinical manifestations of VVC. Overall, these results highlight hemolytic potential as an important contributor to virulence and a promising target for therapeutic intervention [7,11,33,34].

In the present study, we demonstrated that quinalizarin exhibits antifungal activity against all tested *C. albicans* isolates, with MIC values ranging from 2 µg/mL to 256 µg/mL. Although its inhibitory effect on hemolysis in three selected strains was relatively modest, reducing activity to 82–93.7% of the control levels, the treatment with quinalizarin resulted in a significant downregulation of *ECE1* expression to 23–73% of the control levels (untreated cells). These findings suggest that quinalizarin may interfere with regulatory networks controlling virulence gene expression, even when phenotypic inhibition of hemolysis is limited. This indicates that the compound acts not only as a direct antifungal agent but also as a modulator of virulence pathways. Furthermore, the observed synergistic interaction between quinalizarin and fluconazole highlights the therapeutic potential of combining anti-virulence and fungistatic mechanisms to enhance efficacy while potentially mitigating the emergence of antifungal resistance.

Isolates lacking baseline hemolytic activity were included in the study because quinalizarin exhibits broad antifungal and anti-virulence effects on *Candida*, affecting both growth and multiple virulence traits [18]. Their inclusion reflects the natural heterogeneity of clinical *C. albicans* populations and allows for a more comprehensive evaluation of quinalizarin’s activity beyond a single virulence parameter.

Recent studies have identified several additional strategies aimed at disrupting the production or activity of candidalysin. One such approach involves methoxy-apo-enterobactin, a metabolite isolated from *Streptomyces ambofaciens* CJD34, which has been shown to specifically inhibit *ECE1* expression in *C. albicans* and to reduce fungal colonization in murine infection models. These findings highlight the potential of microbial secondary metabolites as a valuable source of natural inhibitors targeting candidalysin-mediated pathogenicity [35]. Additionally, the purinergic receptor antagonist pyridoxal phosphate-6-azophenyl-2′,4′-disulfonic acid has been shown to significantly diminish candidalysin-induced hemolysis by inhibiting its intercalation into synthetic lipid membranes [7]. Furthermore, co-targeting candidalysin alongside other well-established *C. albicans* virulence factors, including adhesins and hydrolytic enzymes, may constitute a particularly effective combination strategy with enhanced therapeutic potential [36]. Another innovative strategy employs anti-candidalysin nanobodies, which exhibit potent neutralizing activity against both synthetic and secreted forms of the toxin. In epithelial cell models, these nanobodies markedly attenuate cytotoxicity, cytokine release, and neutrophil activation, thereby mitigating the hyperinflammatory response characteristic of symptomatic VVC. When used in combination with fluconazole, they display additive effects, simultaneously restricting fungal growth and limiting host tissue damage [37]. A further promising direction involves the development of vaccines targeting candidalysin, designed to elicit protective immune responses analogous to those induced by the NDV-3A vaccine, which targets Als3p [38,39,40].

The present results broaden the understanding of *C. albicans* pathogenicity in VVC, particularly regarding strain-dependent variability in virulence traits associated with the *ECE1* gene and its cytolytic product, candidalysin. As reported by Liu et al. (2021), most models of *C. albicans* pathogenesis rely on the reference strain SC5314; however, natural clinical isolates, such as strain 529L, exhibit distinct pathogenic behaviors that may better reflect mucosal infection dynamics [41]. The studies have shown that, despite comparable *ECE1* and *KEX2* expression levels, *C. albicans* 529L induces markedly lower inflammatory responses and epithelial damage than SC5314. This reinforces the concept that *ECE1* transcript abundance alone does not determine virulence and that post-translational processing and secretion efficiency of candidalysin play decisive roles in pathogenicity. Notably, environmental factors such as the acidic vaginal pH may further modulate hyphal growth and candidalysin secretion; however, *C. albicans* can locally alkalinize its microenvironment through amino acid catabolism, enabling toxin activity even under otherwise inhibitory conditions. Thus, the attenuated phenotype associated with 529L-like alleles may represent an evolutionary adaptation toward reduced virulence and enhanced mucosal persistence, consistent with the commensal nature of many vaginal *C. albicans* strains [12,41,42]. Such inter-strain variability likely contributes to the wide range of hemolytic activities observed in the isolates. These findings provide a basis for future studies to experimentally link hemolytic activity, candidalysin levels, and clinical outcomes, clarifying the contribution of this toxin to VVC pathogenesis.

## 5. Conclusions

In conclusion, this study provides new insights into the pathogenic mechanisms of *C. albicans* in VVC, emphasizing the role of hemolysis as a key virulence determinant. The findings demonstrate significant strain-dependent variability in hemolytic activity among vaginal isolates, highlighting that virulence in *C. albicans* is not solely dictated by *ECE1* expression levels but also by the efficiency of candidalysin processing and secretion. These results support the hypothesis that differential hemolytic potential may contribute to the spectrum of clinical manifestations observed in VVC, although direct clinical correlations were not assessed in this study. Furthermore, we identified quinalizarin as a compound with multifaceted antifungal and anti-virulence properties. Although its direct inhibition of hemolysis was modest, quinalizarin significantly downregulated *ECE1* expression and displayed synergistic activity with fluconazole, underscoring its potential as a therapeutic adjuvant. These findings suggest that targeting virulence pathways rather than fungal viability may represent a promising strategy to reduce host tissue damage, limit inflammation, and mitigate antifungal resistance, though further in vivo validation is required. Future studies should further elucidate the molecular mechanisms regulating candidalysin secretion and evaluate the clinical applicability of combination therapies that integrate conventional antifungals with targeted anti-virulence agents such as quinalizarin.

## Figures and Tables

**Table 1:** Source, hemolysis percentage, and efficiency of hemolytic factor production by strains of *C. albicans*.

Strain No	Source of Strain	Hemolysis (%)	Efficiency of Hemolytic Factor/10^7^ Cells (%)
1	*C. albicans* ATCC 10231	46 ± 4.3	57.5
2	*C. albicans* ATCC 90028	96.8 ± 2.8	121
3	*C. albicans* ATCC 90029	35.4 ± 3.7	39.3
4	Clinical isolate 141	71.4 ± 3.5	79.3
5	Clinical isolate 138	0	0
6	Clinical isolate 102	1.5 ± 1	1.7
7	Clinical isolate 103	21.5 ± 2.3	26.9
8	Clinical isolate 123	9.3 ± 2.2	10.9
9	Clinical isolate 10	2 ± 1	2.8
10	Clinical isolate 144	63.3 ± 6.1	71
11	Clinical isolate 38	0	0
12	Clinical isolate 34	0	0
13	Clinical isolate 14	10.3 ± 2.7	12.8
14	Clinical isolate 94	0	0
15	Clinical isolate 22	0	0
16	Clinical isolate 111	0	0
17	Clinical isolate 98	12.1 ± 4.5	17.3
18	Clinical isolate 112	47.1 ± 2.5	59
19	Clinical isolate 26	0	0
20	Clinical isolate 149	4.7 ± 1.6	6.7
21	Clinical isolate 115	0	0
22	Clinical isolate 100	37 ± 3.3	52.8
23	Clinical isolate 27	0	0
24	Clinical isolate 106	54.4 ± 4.6	68
25	Clinical isolate 39	21.6 ± 2.7	30.8
26	Clinical isolate 9	99 ± 2.8	123.7
27	Clinical isolate 105	0	0
28	Clinical isolate 41	57.7 ± 6.1	72.1
29	Clinical isolate 92	59.8 ± 5.2	85.4
30	Clinical isolate 15	0	0
31	Clinical isolate 13	0	0
32	Clinical isolate 101	40.2 ± 1.8	57.4
33	Clinical isolate 117	36.7 ± 2.1	45.8
34	Clinical isolate 35	36.5 ± 3.1	40.6
35	Clinical isolate 90	0	0
36	Clinical isolate 121	0	0
37	Clinical isolate 6	78.3 ± 4.5	97.8
38	Clinical isolate 132	64.5 ± 2.9	92.1
39	Clinical isolate 191	74.3 ± 2.8	82.6
40	Clinical isolate 134	58 ± 4.7	82.8
41	Clinical isolate 104	17.5 ± 3.3	21.8
42	Clinical isolate 110	16.2 ± 1.6	23.1
43	Clinical isolate 142	59.8 ± 2	66.4
44	Clinical isolate 109	20.4 ± 3.5	25.5
45	Clinical isolate 36	91.2 ± 7.5	114
46	Clinical isolate 124	34.9 ± 3.2	49.8
47	Clinical isolate 46	8.6 ± 3.1	10.7
48	Clinical isolate 116	71.9 ± 8.4	102.7
49	Clinical isolate 93	23.7 ± 4.5	26.3
50	Clinical isolate 137	4 ± 1.3	4.4
51	Clinical isolate 23	33.2 ± 4	49.5
52	Clinical isolate 77	33.6 ± 2.3	42
53	Clinical isolate 143	63.6 ± 4.3	94.9
54	Clinical isolate 128	99 ± 7.6	141.4
55	Clinical isolate 99	98.9 ± 4.5	140.3
56	Clinical isolate 76	0	0
57	Clinical isolate 97	68 ± 5.6	85
58	Clinical isolate 8	8.7 ± 2.1	9.6
59	Clinical isolate 119	4.8 ± 2.2	6.1
60	Clinical isolate 54	81.5 ± 6.3	90.5
61	Clinical isolate 20	62.7 ± 4.3	89.6
62	Clinical isolate 70	8.1 ± 2	10.2
63	Clinical isolate 91	74.3 ± 6.7	82.6
64	Clinical isolate 51	15.9 ± 3.3	22.7
65	Clinical isolate 118	18.4 ± 3.4	23
66	Clinical isolate 37	60.7 ± 3.1	86.7
67	Clinical isolate 114	7.9 ± 1.4	9.9
68	Clinical isolate 130	3 ± 1.6	3.3
69	Clinical isolate 120	29.2 ± 3	36.5
70	Clinical isolate 55	2.9 ± 1.1	4.1
71	Clinical isolate 140	19.3 ± 2.1	24.2
72	Clinical isolate 133	4.3 ± 1.9	6.2
73	Clinical isolate 135	14.8 ± 1.5	16.4
74	Clinical isolate 139	63.7 ± 4.5	98
75	Clinical isolate 129	25.6 ± 3.3	28.4
76	Clinical isolate 74	99 ± 4.9	142
77	Clinical isolate 64	0	0
78	Clinical isolate 24	28.4 ± 3.4	40.6
79	Clinical isolate 152	12.7 ± 1.9	15.8
80	Clinical isolate 89	34.5 ± 2.8	53
81	Clinical isolate 32	70.4 ± 3.8	108.3
82	Clinical isolate 95	34.1 ± 3.2	48.7
83	Clinical isolate 87	22.5 ± 2.1	41
84	Clinical isolate 25	90.2 ± 4.2	138.8
85	Clinical isolate 66	98 ± 3.1	163.3
86	Clinical isolate 86	99.2 ± 2.1	141.7
87	Clinical isolate 73	0	0
88	Clinical isolate 108	20.1 ± 2.6	28.7
89	Clinical isolate 122	19.7 ± 1.6	24.6
90	Clinical isolate 72	99.7 ± 2.9	166.2
91	Clinical isolate 58	76.5 ± 5.1	95.6
92	Clinical isolate 81	4.6 ± 1.1	5.7
93	Clinical isolate 50	94.8 ± 4.8	135.4
94	Clinical isolate 45	58.8 ± 3.9	73.5
95	Clinical isolate 126	0	0
96	Clinical isolate 40	99.5 ± 4.2	153
97	Clinical isolate 75	0	0

**Table 2:** Minimum inhibitory concentrations (MICs) of quinalizarin against reference and clinical *C. albicans* strains.

Strain No	Source of Strain	MIC (µg/mL)
1	*C. albicans* ATCC 10231	8
2	*C. albicans* ATCC 90028	16
3	*C. albicans* ATCC 90029	32
4	Clinical isolate 141	16
5	Clinical isolate 138	8
6	Clinical isolate 102	8
7	Clinical isolate 103	16
8	Clinical isolate 123	64
9	Clinical isolate 10	32
10	Clinical isolate 144	4
11	Clinical isolate 38	32
12	Clinical isolate 34	64
13	Clinical isolate 14	64
14	Clinical isolate 94	4
15	Clinical isolate 22	4
16	Clinical isolate 111	8
17	Clinical isolate 98	128
18	Clinical isolate 112	128
19	Clinical isolate 26	16
20	Clinical isolate 149	8
21	Clinical isolate 115	16
22	Clinical isolate 100	32
23	Clinical isolate 27	32
24	Clinical isolate 106	4
25	Clinical isolate 39	128
26	Clinical isolate 9	16
27	Clinical isolate 105	32
28	Clinical isolate 41	256
29	Clinical isolate 92	256
30	Clinical isolate 15	32
31	Clinical isolate 13	16
32	Clinical isolate 101	16
33	Clinical isolate 117	8
34	Clinical isolate 35	8
35	Clinical isolate 90	4
36	Clinical isolate 121	8
37	Clinical isolate 6	128
38	Clinical isolate 132	8
39	Clinical isolate 191	32
40	Clinical isolate 134	16
41	Clinical isolate 104	32
42	Clinical isolate 110	16
43	Clinical isolate 142	4
44	Clinical isolate 109	16
45	Clinical isolate 36	64
46	Clinical isolate 124	32
47	Clinical isolate 46	32
48	Clinical isolate 116	256
49	Clinical isolate 93	16
50	Clinical isolate 137	8
51	Clinical isolate 23	32
52	Clinical isolate 77	16
53	Clinical isolate 143	32
54	Clinical isolate 128	128
55	Clinical isolate 99	256
56	Clinical isolate 76	4
57	Clinical isolate 97	16
58	Clinical isolate 8	8
59	Clinical isolate 119	2
60	Clinical isolate 54	256
61	Clinical isolate 20	32
62	Clinical isolate 70	16
63	Clinical isolate 91	2
64	Clinical isolate 51	16
65	Clinical isolate 118	8
66	Clinical isolate 37	16
67	Clinical isolate 114	32
68	Clinical isolate 130	8
69	Clinical isolate 120	64
70	Clinical isolate 55	256
71	Clinical isolate 140	32
72	Clinical isolate 133	8
73	Clinical isolate 135	16
74	Clinical isolate 139	8
75	Clinical isolate 129	32
76	Clinical isolate 74	256
77	Clinical isolate 64	2
78	Clinical isolate 24	16
79	Clinical isolate 152	32
80	Clinical isolate 89	32
81	Clinical isolate 32	8
82	Clinical isolate 95	32
83	Clinical isolate 87	32
84	Clinical isolate 25	128
85	Clinical isolate 66	16
86	Clinical isolate 86	256
87	Clinical isolate 73	64
88	Clinical isolate 108	16
89	Clinical isolate 122	64
90	Clinical isolate 72	32
91	Clinical isolate 58	32
92	Clinical isolate 81	16
93	Clinical isolate 50	32
94	Clinical isolate 45	8
95	Clinical isolate 126	4
96	Clinical isolate 40	256
97	Clinical isolate 75	2

**Table 3:** Synergistic interactions between fluconazole and quinalizarin against selected *C. albicans* strains determined by the fractional inhibitory concentration index (FICI).

*Candida* Strain	MIC Fluconazole (µg/mL)	MIC Quinalizarin (µg/mL)	FIC_A_	FIC_B_	FICI (Interpretation)
*C. albicans* ATCC 10231	256	8	0.031	0.25	0.281 (Synergism)
*C. albicans* ATCC 90028	64	16	0.0156	0.25	0.265 (Synergism)
*C. albicans* isolate 50	8	32	0.031	0.015	0.046 (Synergism)

Abbreviations: MIC, minimal inhibitory concentration; FIC_A_, fractional inhibitory concentration of compound A (fluconazole); FIC_B_, fractional inhibitory concentration of compound B (quinalizarin); FICI, fractional inhibitory concentration index.

## Data Availability

All data supporting the results reported in this article are included within the article.
